# Can We ‘Read’ the Eye-Movement Patterns of Readers? Unraveling the Relationship Between Reading Profiles and Processing Strategies

**DOI:** 10.1007/s10936-016-9418-2

**Published:** 2016-03-21

**Authors:** Arnout Koornneef, Iris Mulders

**Affiliations:** 1 Institute of Education and Child Studies, Leiden University, Pieter de la Court gebouw, Wassenaarseweg 52, 2333 AK Leiden, The Netherlands; 2 Utrecht Institute of Linguistics OTS, Utrecht University, Utrecht, The Netherlands

**Keywords:** Eye-movement patterns, Reading strategies, Implicit causality, Individual differences, Anticipation

## Abstract

In an eye-tracking experiment we examined the risky reading hypothesis, in which long saccades and many regressions are considered to be indicative of a proactive reading style (Rayner et al. in Psychol Aging 21(3):448, 2006; Psychol Aging 24(3):755, 2009). We did so by presenting short texts—that confirmed or disconfirmed verb-based implicit causality expectations—to two types of readers: proactive readers (long saccades, many regressions) and conservative readers (short saccades, few regressions). Whereas proactive readers used implicit causality information to predict upcoming referents, and slowed down immediately when they encountered a pronoun that was inconsistent with these verb-based expectations, the conservative readers slowed down much later in the sentence. These findings were consistent with the predictions of the risky reading hypothesis and as such presented novel evidence for the general idea that the eye-movement profile of readers reveals valuable information about their processing strategy.

## Introduction

Reading is a complex skill that requires a precisely timed interplay between numerous perceptual, linguistic and general cognitive mechanisms. To name just a few, readers make extensive use of their visual system, they constantly access various long term memory systems, they routinely store (partially) processed information in working memory, and they monitor their behavior through various executive and control functions. Given that reading involves such a diverse array of perceptual and cognitive (sub)skills, it is not surprising that people differ in the way they process written text. Evidently, they diverge on coarse dimensions such as reading speed and reading proficiency. However, they also tend to differ in more subtle ways. The way people move their eye gaze across a text, for example, varies among individuals. Whereas in some cases readers make an eye fixation on every single word, others skip words frequently by making long saccades. Furthermore, some readers tend to read almost exclusively in a linear and progressive manner, yet other types of readers make a lot of regressive eye movements, going back and forth in a text regularly (e.g., Hyönä et al. 2002; Hyönä and Nurminen 2006; Rayner et al. 2006, 2009).

A question that has received some attention in the literature is whether these individual differences in eye-movement patterns are informative about how readers process a text. For example, Hyönä et al. (2002) analyzed the eye movements of university students and were able to identify various reading profiles (cf., Goldman and Saul 1990; Hyönä and Nurminen 2006). They proposed labels like linear readers, topic structure processors and non-selective reviewers to describe these profiles. The linear readers typically did not look back to previous sentences. In contrast, the topic structure processors and the non-selective reviewers returned frequently to earlier parts of the text. The crucial difference between these two latter groups was that topic structure processors showed sensitivity to the text’s content structure—they looked back to the main points of the text.

In another series of eye-tracking studies, Rayner et al. (2006, 2009) took a different approach. They focused on two of the most basic aspects of eye movements to assess reading strategies: the distance and the direction of the saccades that people make between fixations. In a number of experiments with younger and senior adults, they observed that senior adult readers make longer saccades, skip words more frequently and show more backward eye movements. This pattern was interpreted as the result of a subconscious processing strategy in which senior readers make frequent guesses about the continuation of an unfolding sentence. Rayner et al. postulated the label *risky reading* to depict this proactive eye-movement profile (in the present study the labels risky reading and *proactive reading* are used interchangeably).

This latter series of studies illustrates an interesting direction in reading research: individual differences in global eye-movement patterns are thought to illuminate differences in processing strategies pertaining to the reader’s recruitment of anticipatory comprehension mechanisms. However, the empirical findings in support of this hypothesis are scarce. In the present study we therefore aim at providing further evidence for this potentially fruitful framework by investigating whether differences in eye-movement profiles are reflected in the way readers use the meaning of *implicit causality verbs* while constructing a mental representation of a short discourse. As discussed in more detail below, implicit causality information is used by readers to generate (referential) expectations about the continuation of a discourse. As such it offers a unique window into the anticipatory features of the human language system (cf., Pyykkönen and Järvikivi 2010; Van Berkum et al. 2007, 2013), and more specifically, allows a fine-grained examination of the relationship between reading profiles and (proactive) processing strategies.

### Implicit Causality Verbs

Implicit causality is a property of a group of interpersonal verbs in which one or the other of the verb’s arguments is implicated as the underlying cause of the action or attitude (e.g., Au 1986; Garvey and Caramazza 1974; Garvey et al. 1975; Greene and McKoon 1995; Koornneef and Van Berkum 2006; Long and De Ley 2000; Stewart et al. 2000). For example, in a sentence like ‘David apologized to Linda$\ldots$’ the verb *apologize* creates a bias towards the first noun phrase (NP1). It is more likely that the sentence will continue about David (e.g., because he had forgotten to mail the letter). In contrast, in a sentence fragment like ‘David praised Linda$\ldots$’ the bias of the verb *praise* is towards the second noun phrase (NP2; e.g., because she had done well). Hence, verbs such as *praise* and *apologize* are associated with different referential biases that either render NP1 or NP2 a privileged status in the text.

Implicit causality verbs present an interesting test case for the hypothesis that differences in reading patterns correspond to differences in reading strategies, for several reasons. First, we already mentioned that anticipatory mechanisms are known to play an important role in the activation and usage of implicit causality information (e.g., Featherstone and Sturt 2010; Koornneef and Sanders 2013; Koornneef and Van Berkum 2006; McDonald and MacWhinney 1995; Pyykkönen and Järvikivi 2010; Van Berkum et al. 2007). For example, in short discourses such as ‘David praised Linda. He was impressed with her achievement.’, readers anticipate that the text will continue about Linda (not David) and consequently they slow down when they encounter the pronoun *he*—this finding will be referred to as the pronoun-inconsistency effect. Second, this influence of implicit causality on pronoun resolution is a very robust phenomenon and has been replicated across various languages and methodologies (e.g., Cozijn et al. 2011; Featherstone and Sturt 2010; Koornneef and Van Berkum 2006; Long and De Ley 2000; Pyykkönen and Järvikivi 2010; Van Berkum et al. 2007). Third, there is preliminary evidence that the usage of implicit causality information is mediated by the processing strategy of the reader. More specifically, in an ERP-experiment, Van Berkum et al. (2013) investigated how people’s mood affected the proactive use of implicit causality. Although the main aim of their study was not to decipher the specifics of implicit causality, but rather, to explore how language processing is embodied into other systems of the human brain, the results were striking. Whereas people in a good mood displayed verb-bias inconsistency effects right at the pronoun, the influence of implicit causality was absent for people in a bad mood. According to Van Berkum et al., one of the explanations for this contrast could be related to the willingness of the participants to use implicit causality information to anticipate upcoming referents. In their view, readers in a good mood were more likely to use a proactive, (i.e., top-down, predictive) processing strategy and, hence, used implicit causality information to predict that a story would continue about a particular referent. In a bad mood, however, the same readers adopted a more retroactive (i.e., bottom-up, conservative) processing strategy, and displayed no early sensitivity to the information offered by implicit causality verbs.

### The Present Study

In a broad sense the goal of the present study was to further explore the idea that the eye-movement signature of readers reveals information about their processing strategy. We did so by examining the risky reading hypothesis (Rayner et al. 2006, 2009) in which long saccades and many regressions are considered to be indicative of a predictive reading style. In light of the recent proposal that only people in a good mood, and consequently proactive processing mode, use verb-based implicit causality to anticipate upcoming referents (Van Berkum et al. 2013), we made the conjecture that corresponding findings should be observed in terms of the risky reading hypothesis: only readers displaying a proactive reading style (long saccades and many regressions) should be using implicit causality information to anticipate the referential continuation of a story.

This prediction was tested in an eye-tracking experiment with young adults (university students) as participants. They read short Dutch stories in which the subject pronoun of the final sentence was either consistent or inconsistent with the implicit causality bias of the main verb of the preceding sentence (see Table 1). The eye movement recordings were utilized in two ways. First, the data was used to determine the reading profile of the participants. Based on the idea that proactive readers make longer saccades and look back more often in a text, these dimensions were used to assign the participants to two groups displaying ‘opposite’ reading patterns. More specifically, the first group consisted of readers that made relatively long saccades and many regressions, i.e., the proactive readers. The second main group consisted of readers that displayed a contrasting pattern. They made short saccades and few regressions. We use the term *conservative* to refer to this type of readers as they tend to avoid regressive eye-movements—which are often associated with processes of reanalysis and repair.

**Table 1:** NP1-biased example of stimuli

Introductory sentences	David en Linda reden allebei behoorlijk hard. Bij een druk kruispunt botsten zij met hun auto’s stevig op elkaar
	(David and Linda were both driving pretty fast. At a busy intersection they crashed hard into each other)
Critical sentences	
Consistent condition	$\underline{David}$ bood zijn excuses aan Linda aan. *Hij* was volgens de getuigen van het ongeluk de veroorzaker van alle ellende
	($\underline{David}$ apologized to Linda. *He* according to the witnesses was the one to blame)
Inconsistent condition	$\underline{\hbox {Linda}}$ bood haar excuses aan *David* aan. *Hij* was volgens de getuigen van het ongeluk niet de veroorzaker van alle ellende
	($\underline{\hbox {Linda}}$ apologized to *David*. *He* according to the witnesses was not the one to blame)

The verb has a bias towards the subject, i.e., the underlined story character. The story character in italics is the referent of the pronoun

In addition, we used the eye-tracking data to compute commonly reported reading time measures to assess the processing costs for the readers at—and immediately after—the critical pronoun. Based on the general hypothesis that different reading profiles correspond to different reading strategies, we predicted that the time course of implicit causality should vary across the two reading groups. That is, if proactive readers use implicit causality information to anticipate that the story will continue about a particular referent, we should be able to detect the pronoun-inconsistency effect at the earliest moment possible, i.e., right at the critical pronoun. In contrast, for the conservative readers we expected no pronoun-inconsistency effect at all, or alternatively, a delayed effect. This delayed influence was deemed plausible, because even though readers may not be using implicit causality information to anticipate upcoming referents, they may use the information to interpret the pronoun retroactively (Garnham et al. 1996; Stewart et al. 2000). Since the (re)activation of implicit causality and the integration of the pronoun do not occur instantaneously, the associated processing costs typically spill over to subsequent words (cf., Featherstone and Sturt 2010; Koornneef and Van Berkum 2006). As a result, any significant pronoun-inconsistency effects for the conservative readers should emerge later in the sentence.

## Methods

### Participants

Participants were 46 undergraduate students (39 female, mean age 23, range 18–49 years) who received money for their participation. They were native speakers of Dutch, without a diagnosed reading or learning disability and with normal or corrected to normal vision.

### Materials

The stimuli were created by adapting the stimuli of Koornneef and Sanders (2013). The stimulus set contained 48 items. For each item, there was a consistent and inconsistent condition (i.e., in all there were 96 unique stories). To control for the distance between the pronoun and antecedent, first mention, and the antecedent’s structural position (see Garnham 2001, for an overview of the relevance of these factors), half of the items were constructed around implicit causality verbs with a bias towards NP1, and half of the items were constructed around implicit causality verbs with a bias towards NP2. Koornneef and Sanders (2013) assessed the strength of the overall bias of these items in a paper-and-pencil cloze-task. In 71 % of the cases, people continued the story with the biased argument of the implicit causality verbs.

Examples of the two versions of the Dutch items are presented in Table 1, together with their approximate translation. In the first sentence a situation was sketched in which a man and a woman were introduced by name. In the second sentence a pronominal (usually ‘zij’, *they*) was used to keep both characters in focus to an equal extent. The third sentence contained the implicit causality verb and repeated the names of the two characters. The fourth sentence started with the critical pronoun ‘hij’ (*he*). Instead of contrasting the Dutch equivalents of *he* and *she*, we manipulated whether the fixed pronoun ‘hij’ was consistent with the verb’s bias by swapping the argument position of the man and the woman involved. The Dutch equivalent of *she* was avoided, because it is ambiguous between a singular and a plural third-person pronoun.

To be able to (i) get a fine-grained measure of the time course of implicit causality, (ii) interpret potential delayed effects, and (iii) accommodate for general spillover, at least five words after *he* were held constant across conditions. After these five words the consistent and inconsistent versions diverged, and ended with explicit information that made the story coherent as a whole.

The stimuli were divided into two lists, with only one version of each item in a particular list. Twenty-four stories of a different study examining the processing costs of various coherence relations were included as fillers. These filler items involved a mix of temporal and (forward) causal coherence relations (e.g., ‘Mary jumped on the table. The table broke. She apologized and took a seat on the couch.’). The participants were assigned to one of the lists, and for each participant the list was presented in a unique pseudo-randomized order. Half of the experimental and filler trials were followed by a statement about the story to encourage discourse comprehension. Participants had to indicate whether the statement about the story was correct or false (half were correct and half were false). These statements never directly probed the interpretation of the pronoun. All participants scored above 80 % correct ($M=$ 92 %).

### Procedure

Eye movements were recorded with a desktop-mounted EyeLink 1000 eye tracker, which samples the eyes at 500 Hz. The system has an eye position tracking range of 32$^{\circ }$ horizontally and 25$^{\circ }$ vertically, with a gaze position accuracy of 0.5$^{\circ }$. Viewing was binocular but the tracker monitored only the gaze location of the right eye. All participants were individually tested in a sound-treated booth at Utrecht University. Each session started with a written and oral instruction, after which the eye tracker was calibrated. Upon successful calibration the experiment started with five practice trials. The stories were presented in their entirety on a CRT screen at a viewing distance of approximately 60 cm. Before presentation, a fixation mark appeared on the screen at the position of the first word of the first sentence. Participants were instructed to fixate this mark and after successful fixation the story appeared automatically. Participants pressed a button to progress when they finished reading a story. The comprehension questions were answered using two buttons on the same response box. Each session consisted of four reading blocks. In between blocks, the readers were allowed a short break, after which the eye-tracker was recalibrated. A full session was completed within 45 min (with a maximum time-on-task of 30 min).

## Results

### Categorizing Proactive and Conservative Readers

First we determined the reading profile of each participant by computing the average distance of their saccades and by computing the average probability that they made a regressive eye movement. The average saccade distance was based on the distance of all first pass progressive saccades before readers entered the critical pronoun region. We did not include data points of the other sections of the text as it would confound the results—i.e., readers that slow down or regress more often from the inconsistent pronoun onwards, will have a higher probability of being characterized as proactive readers, which in turn increases the probability that the predictions of the risky reading hypothesis would be confirmed. To reduce noise, we did not include the first three saccades of a trial. In addition, we removed saccades that preceded or followed a blink, and saccades preceding or following an unassigned fixation (i.e., a fixation that fell outside the layout of the text). Furthermore, return sweeps and the first progressive saccade after a regression were not included. A similar procedure was followed for computing the average probability of a regression for each participant: we only considered regressions during first pass reading before readers entered the critical regions; the first three fixations of a trial were excluded; regressive eye-movements containing blinks, or directly following a blink or an unassigned fixation were excluded; return sweeps and regressive eye-movements directly following a return sweep were excluded.

We created the two main groups of readers (proactive and conservative readers) by conducting a ‘dual’ median-split procedure: Every participant received either the label ‘long’ or ‘short’ for the measure *saccade distance*, and the label ‘many’ or ‘few’ for the measure *probability of a regression*. Group membership was defined as a combination of these two labels. Proactive readers made long saccades and many regressions ($N =15$; saccade distance in number of letters: $M =9.6$, $\textit{SE} =0.26$; probability of a regression: $M=0.23$, ${\textit{SE}}=0.01$). Conservative readers made short saccades and few regressions ($N =15$; saccade distance: $M=7.5$, ${\textit{SE}}=0.17$; probability of a regression: $M=0.11$, ${\textit{SE}}=0.01$).

Due to the nature of the median split procedure, two other (smaller) groups of readers remained. A group of *fast* readers made long saccades and few regressions ($N=8$; saccade distance: $M=9.2$, ${\textit{SE}}=0.30$; probability of a regression: $M=0.13$, ${\textit{SE}}=0.01$). A final group of *slow* readers made short saccades and many regressions ($N=8$; saccade distance: $M=7.8$, ${\textit{SE}}=0.15$; probability of a regression: *M* = 0.22, ${\textit{SE}} = 0.02$).^1^

### Dependent Variables

To examine the predicted differences in the time course of implicit causality, we computed commonly reported reading time measures for the regions of interest—i.e., the critical pronoun and the five subsequent words. *First fixation duration *reflects the duration of the very first fixation on a word. *First gaze duration* is the sum of all fixation durations on a word or region before the reader either moves on, or looks back into the text. *Right bound durations* sum up all fixations within a region before moving on progressively. This measure includes fixations that follow regressions. *Regression path durations* are the sum of fixation durations from the time when the reader enters a region, to the time when the reader enters the following region. This means that if the reader looks back after reading a particular region, the regression path time includes all fixation durations of this regression (see Table 2 for the means of the reading time measures).^2^

**Table 2:** Mean reading times and skipping rates for the four reading groups at the critical pronoun and the five subsequent words

	Sentence region											
	Pronoun		Pro $+$ 1		Pro $+$ 2		Pro $+$ 3		Pro $+$ 4		Pro $+$ 5	
	Mean	SE	Mean	SE	Mean	SE	Mean	SE	Mean	SE	Mean	SE
*First fixation duration*												
Proactive												
con	172	7	231	6	220	5	214	5	208	4	182	5
inc	182	8	238	6	231	8	221	5	208	5	198	5
Conservative												
con	213	7	226	5	226	6	209	4	197	4	176	5
inc	220	9	221	4	223	6	222	5	197	4	187	5
Fast												
con	175	9	215	4	203	7	190	4	186	5	177	7
inc	188	11	217	5	211	7	208	6	195	5	174	7
Slow												
con	250	18	262	9	221	10	220	5	214	6	191	7
inc	220	20	269	15	235	10	222	7	201	6	195	8
*First gaze duration*												
Proactive												
con	172	7	230	6	227	7	215	5	222	5	187	6
inc	187	7	239	7	236	8	226	6	221	6	198	5
Conservative												
con	212	8	229	5	227	5	216	5	214	5	180	5
inc	223	9	223	4	238	8	232	6	223	6	207	7
Fast												
con	190	17	217	4	212	8	191	5	198	6	186	8
inc	188	11	216	5	222	9	213	7	222	8	182	9
Slow												
con	250	18	266	11	220	9	235	8	259	13	195	7
inc	220	20	283	17	243	12	239	8	219	9	202	9
*Right bound duration*												
Proactive												
con	172	7	231	6	233	7	235	7	242	7	190	7
inc	195	11	237	7	244	9	254	9	233	6	211	6
Conservative												
con	212	8	233	6	235	6	229	7	226	6	187	6
inc	223	9	232	5	246	8	241	7	233	7	219	8
Fast												
con	190	17	217	4	212	8	189	4	199	6	186	8
inc	194	9	216	5	224	9	220	8	233	9	189	9
Slow												
con	250	18	269	11	251	12	265	11	291	17	212	9
inc	230	21	294	18	258	14	266	10	233	10	218	11
*Regression path duration*												
Proactive												
con	237	33	235	6	280	12	315	16	341	19	228	15
inc	273	35	262	15	314	26	355	25	311	14	268	18
Conservative												
con	231	19	243	7	243	7	241	9	252	11	205	10
inc	237	12	247	8	268	16	258	9	260	11	246	12
Fast												
con	246	44	221	4	232	11	218	11	207	8	197	11
inc	235	27	222	6	242	11	243	12	263	16	208	13
Slow												
con	278	43	292	15	319	22	326	22	399	32	257	17
inc	291	43	292	18	320	22	336	20	278	17	245	18
*Skipping rates*												
Proactive												
con	0.87	0.02	0.56	0.03	0.55	0.03	0.39	0.03	0.27	0.02	0.36	0.03
inc	0.86	0.02	0.56	0.03	0.58	0.03	0.35	0.03	0.30	0.03	0.37	0.03
Conservative												
con	0.78	0.02	0.26	0.02	0.45	0.03	0.47	0.04	0.28	0.03	0.48	0.04
inc	0.75	0.02	0.31	0.03	0.44	0.04	0.46	0.04	0.31	0.04	0.46	0.04
Fast												
con	0.87	0.02	0.27	0.03	0.68	0.03	0.51	0.03	0.28	0.02	0.49	0.03
inc	0.83	0.03	0.37	0.04	0.62	0.03	0.47	0.03	0.33	0.03	0.43	0.03
Slow												
con	0.87	0.03	0.30	0.03	0.55	0.04	0.34	0.04	0.25	0.03	0.38	0.04
inc	0.79	0.03	0.31	0.04	0.51	0.04	0.29	0.03	0.27	0.03	0.39	0.04

Reading times in milliseconds. The values reflect the means with standard error (SE) aggregated over all data points. *Pro* pronoun; *con* consistent condition; *inc* inconsistent condition

Prior to all analyses, 1 % of all trials was removed, because major tracker losses or eye blinks made it impossible to determine the course of fixations. For all different measures, skipped regions were treated as missing data. In addition, reading times more than two standard deviations from both the participant’s mean and the item’s mean in a region in a particular condition were excluded from the analysis ($<$15 % for all measures).

### Main Analyses: Proactive Readers Versus Conservative Readers

The main prediction in the current study was that proactive readers should display a very early influence of implicit causality (i.e., right at the pronoun), yet conservative readers should display a delayed, or no influence of implicit causality. To test this prediction, separate mixed-effects analyses were conducted for all regions of interest (i.e., the critical pronoun and the five subsequent words).^3^ The dependent variables were log transformed to correct for right skewness. Since it is not clear how to determine the degrees of freedom for the *t* statistics estimated by the mixed models for continuous dependent variables (Baayen et al. 2008), we do not report degrees of freedom and *p* values. Instead, statistical significance at approximately the 0.05 level is indicated by values of the *t* statistics $\ge$1.96 (see e.g., Schotter et al. 2014). Unless mentioned otherwise, we only discuss effects that satisfied this criterion. The models tested the main and interaction effects of the fixed factors consistency (two levels: consistent pronoun, inconsistent pronoun) and group (two levels: proactive, conservative), and included the maximal participant and item random-effect structure that resulted in a converging model (Barr et al. 2013). Initial models included the consistent pronoun condition for the proactive reading group as reference level. When necessary (e.g., to interpret interaction effects), analogous models were applied with the consistent pronoun condition for the conservative reading group as reference level.

At the critical pronoun, the analyses returned a significant Consistency $\times$ Group interaction for the right bound durations ($\beta = -0.17$, ${\textit{SE}} = 0.083$, $t = -2.0$). More specifically, whereas the proactive readers displayed a pronoun inconsistency effect ($\beta = 0.13$, ${\textit{SE}} = 0.065$, $t = 2.0$), the conservative readers did not display a reading time difference between the consistent and inconsistent pronouns ($\beta = -0.021$, ${\textit{SE}} = 0.048$, $t = -0.4$).

Five words after the critical pronoun, a different pattern was observed in the right bound durations. Although the Consistency $\times$ Group interaction was not significant ($\beta = 0.056$, ${\textit{SE}} = 0.060$, $t = 0.94$), the inconsistency effect seemed more pronounced for the conservative readers. Whereas conservative readers displayed a significant inconsistency effect ($\beta = 0.12$, ${\textit{SE}} = 0.040$, $t = 3.1$), this effect fell short of significance for the proactive readers ($\beta = 0.067$, ${\textit{SE}} = 0.044$, $t = 1.5$). The same pattern was observed five words after the pronoun in the first gaze and regression path measures. The Consistency $\times$ Group interactions were not reliable (first gaze: $\beta = 0.075$, ${\textit{SE}} = 0.057$, $t = 1.3$; regression path: $\beta = 0.060$, ${\textit{SE}} = 0.075$, $t = 0.80$), yet the inconsistency effects seemed more pronounced for the conservative readers (first gaze: $\beta = 0.11$, ${\textit{SE}} = 0.038$, $t = 2.8$; regression path: $\beta = 0.16$, ${\textit{SE}} = 0.051$, $t = 3.1$) than for the proactive readers (first gaze: $\beta = 0.031$, ${\textit{SE}} = 0.041$, $t = 0.76$; regression path: $\beta = 0.097$, ${\textit{SE}} = 0.054$, $t = 1.77$).

Overall, these results were consistent with the main prediction that proactive readers should display a very early influence of implicit causality, i.e., right at the pronoun, whereas conservative readers should display a delayed (or no) influence of implicit causality.

### Exploratory Analyses: Fast Readers and Slow Readers

Since the categorization of the proactive and conservative readers was based on a dual median split procedure, two other (smaller) groups of readers could be identified: fast readers and slow readers. Given the exploratory nature of the analyses for these fast and slow readers, together with the fact that there was no strong reason to assume that a specific interaction between the two groups should emerge, we studied the time course of implicit causality separately for these remaining reading profiles. All mixed-effects models fitted on the various reading time measures included the fixed effect of Consistency (with the consistent condition as reference level) and the maximal participant and item random-effect structure that resulted in a converging model.^4^

The analysis for the fast readers revealed a significant effect of Consistency three words after the critical pronoun in first fixation duration ($\beta = 0.088$, ${\textit{SE}} = 0.039$, $t = 2.2$), first gaze duration ($\beta = 0.097$, ${\textit{SE}} = 0.046$, $t = 2.1$) and right bound duration ($\beta = 0.13$, ${\textit{SE}} = 0.040$, $t = 3.3$). In addition, significant effects of Consistency emerged four words after the pronoun in right bound duration ($\beta = 0.13$, ${\textit{SE}} = 0.049$, $t = 2.6$) and regression path duration ($\beta = 0.19$, ${\textit{SE}} = 0.067$, $t = 2.9$). Similar to the conservative readers, the fast readers did not slow down immediately due to an inconsistent pronoun, but the effect became visible somewhat later in the sentence.

The analysis for the slow readers revealed a significant effect of Consistency at the pronoun in first fixation duration ($\beta = -0.20$, ${\textit{SE}} = 0.095$, $t = -2.1$) and first gaze duration ($\beta = -0.20$, ${\textit{SE}} = 0.095$, $t = -2.1$). In addition, significant effects of Consistency emerged four words after the pronoun in right bound duration ($\beta = -0.16$, ${\textit{SE}} = 0.058$, $t = -2.8$) and regression path duration ($\beta = -0.26$, ${\textit{SE}} = 0.077$, $t = -3.4$).^5^ However, in contrast to the results for the other reading groups, the observed differences in both regions were in the opposite direction, i.e., reading times were longer after a *consistent* pronoun.

### Skipping Rates of the Pronoun

The critical region in the current study consisted of a short and frequently used word, i.e, the Dutch pronoun ‘hij’ (*he*). As readers tend to ‘skip’ short and highly frequent words during first pass reading—and process these words parafoveally instead (e.g., Rayner 1998)—we examined the skipping rates for the critical pronoun. As reported in Table 2, the pronoun skipping rates were high for all groups of readers (proactive readers, $M=$ 0.86; conservative readers, $M=$ 0.77; fast readers, $M=$ 0.85; slow readers, $M=$ 0.83). To account for potential differences of the skipping rates as a function of the various reading groups and the pronoun consistency manipulation, mixed-effects logistic regression analyses were conducted. Since the primary aim of these analyses was to emphasize the absence of Consistency $\times$ Group interactions—which would complicate the interpretations of the reading time results reported above—we opted for anti-conservative random-intercepts-only models (see Barr et al. 2013).^6^

In the first model we compared the skipping rates of the groups of main interest: Proactive readers and conservative readers. The model tested the main and interaction effects of the fixed factors Group (two levels: proactive, conservative) and Consistency (two levels: consistent pronoun, inconsistent pronoun). None of the main or interaction effects were significant. However, the main effect of Group approached significance: Proactive readers displayed a trend towards higher skipping rates than conservative readers ($\beta = 0.71$, ${\textit{SE}} = 0.39$, $z = 1.8$, $p = .07$).

To explore the potential differences in the skipping rates for the fast and slow readers (in comparison to the proactive and conservative readers), a second logistic regression model was fitted on the data. Instead of two levels, the fixed factor Group now consisted of four levels (proactive readers, conservative readers, fast readers, slow readers). The results showed one main effect, but more importantly, no interaction effects. The significant main effect confirmed the statistical trend of the previous model. Proactive readers skipped the pronoun more often than conservative readers ($\beta = 0.70$, ${\textit{SE}} = 0.34$, $z = 2.0$, $p < .05$). The most straightforward explanation for this difference is that proactive readers made longer saccades in general, thereby increasing the probability of skipping the pronoun.

### Post-hoc Analyses of the Skipping Rates

Although first pass skipping rates of 80 % are not uncommon for very short and highly predictable words (Drieghe et al. 2004), three series of post-hoc control analyses were conducted to minimize the likelihood that the skipping rates would confound the interpretation of the reading time results.

First, due to the influence of parafoveal preview, readers may have regularly interpreted the critical pronoun while fixating the region that preceded the pronoun. Hence, at least part of the pronoun (in)consistency effects could have surfaced before readers actually fixated the pronouns. To account for this possibility, we computed and analyzed the various reading time measures for the sentence region immediately preceding the critical pronoun. The mixed-effects analyses returned neither a significant effect of Consistency, nor a significant Consistency $\times$ Group interaction.^7^ Hence, although it seems unlikely that parafoveal preview did not play a role at all, we are confident that it does not pose problems for our discussion of the reading time results.

Second, since the inconsistency effect for the proactive readers emerged exclusively at the critical pronoun—a region that was skipped frequently—it is possible that proactive readers merely displayed an influence of implicit causality when they happened to have fixated the critical pronoun. Moreover, their sensitivity to implicit causality information in the trials in which the pronoun was skipped remains unclear. As can be seen in Table 2, there appeared to be a tendency towards an inconsistency effect for proactive readers in later sentence regions as well—in particular in the regression path durations. To examine whether reliable inconsistency effects in these later sentence regions were somewhat masked by the trials in which the pronoun was fixated, we ran an additional series of mixed-effects analyses for the proactive readers in which we excluded the trials wherein the pronoun was fixated during first-pass reading. The results showed a significant inconsistency effect in the regression path durations, five words after the critical pronoun ($M_{\mathrm{con} }= 213\,\hbox {ms}, {\textit{SE}}_{\mathrm{con}} = 11\,\hbox {ms}; \hbox {M}_{\mathrm{inc}}= 271\,\hbox {ms}, {\textit{SE}}_{\mathrm{inc}}= 21\,\hbox {ms}$; mixed-effects analysis results: $\upbeta = 0.12$, ${\textit{SE}} = 0.062$, $t = 2.0$). As this region was fixated by the proactive readers in many trials (i.e., the skipping rate in this region was around 37 %), it can be concluded that implicit causality was a relevant factor in most of the trials, even when the pronoun region was skipped.^8^

Third, to avoid referential ambiguity in the experimental items, we exclusively included the male-gendered ‘hij’ (*he*) as the critical pronoun. Despite the fact that the filler items included female-gendered pronouns, this may have allowed participants to develop an anticipatory strategy in which they were able to expect a sentence beginning with ‘he’, irrespective of the preceding materials. This in turn would provide an explanation for the high skipping rates of the pronoun in the current study. To examine this possibility, the skipping rates of the pronoun in the first reading block of the experiment were compared to the skipping rates in the final block (block 4) of the experiment. If the participants developed a task-induced anticipatory strategy—which led them to skip the critical pronoun—the skipping rates in block 4 should be higher than the skipping rates in block 1. This was not the case. In fact, numerically the skipping rate in block 1 (86 %) was higher than in block 4 (82 %). To test whether skipping rates developed differently over the course of the experiment as a function of reading group, a mixed-effects logistic regression was conducted.^9^ The model tested the interaction effects of the fixed factors Group (four levels: proactive readers, conservative readers, fast readers, slow readers) and Block (two levels: block 1, block 4). Since the aim of the analysis was to emphasize the absence of a difference, a random-intercepts-only models was fitted on the data. None of the interactions in the model approached significance, making it less likely that task-induced strategies affected the skipping rates of the pronoun in a different way for the various types of readers.

## Discussion

In line with current directions of reading research, we postulated that readers who display different reading profiles also employ different reading strategies. To test this general hypothesis we used a median split procedure, based on differences in the distance and direction of the saccades of readers, to classify two contrasting types of readers: proactive readers (long saccades, many regressions) and conservative readers (short saccades, few regressions). On the basis of previous work (Rayner et al. 2006, 2009; Van Berkum et al. 2013), the key prediction of the eye-tracking experiment was that a contrast should emerge between proactive and conservative readers in the time course of their usage of implicit causality information. This prediction was borne out by the data. Whereas proactive readers slowed down rapidly when they fixated a bias-inconsistent pronoun, conservative readers slowed down much later, five words further into the sentence.

In addition to proactive and conservative readers, two other reading profiles were identified: slow and fast readers. Although we emphasize that the analyses of these reading profiles were only exploratory in nature, an interesting pattern was observed. Similar to the conservative readers, the fast readers displayed a delayed pronoun-inconsistency effect, emerging three words after the critical pronoun. Furthermore—and arguably more interestingly—for the slow readers a somewhat unexpected pattern was observed. They displayed a reversed pronoun-inconsistency effect, meaning that the *consistent* pronoun induced a processing disadvantage—not the inconsistent pronoun. In other words, for slow readers implicit causality information did not play a role during pronoun resolution, but they seem to be using the meaning of the verb in a different, perhaps even completely opposite manner. We will return to this issue later in our discussion.

Another interesting secondary finding was that readers displayed a high overall skipping rate of the critical pronoun. Whereas previous studies with Dutch materials reported that about half of the pronouns were skipped during first pass reading (e.g., Drieghe et al. 2007; Vonk 1984), four out of five pronouns were skipped in the present study. We cannot rule out unequivocally that these higher skipping rates arose as a side-effect of some methodological choices we made (e.g., exclusively present ‘hij’ as the critical pronoun). Importantly, however, the control analyses in the “Results” section suggest that it is unlikely that the skipping rates were driven by these factors alone.

The question then remains why in the current study the skipping rates were about twice as high as in previous studies. Although this question can be addressed in various ways, at least part of the answer may be related to two crucial differences in the materials of the studies under discussion. First, in contrast to previous studies, the critical pronoun in the current study was the first word of a sentence, and consequently, the first letter of the pronoun was capitalized. This may have functioned as a visual aid, which reduced the need to make a first pass fixation on the pronoun to achieve visual recognition. Second, whereas in the current study the critical sentences were embedded in a larger four-sentence discourse, previous studies presented single sentences—without a clear context. Since the reading pattern of a single sentence may differ substantially from the reading pattern of a larger discourse, this could also potentially explain the discrepancies in skipping rates as observed across studies. In fact, it could be argued that presenting short discourses to the participants, as opposed to isolated sentences, allowed them to approach a more natural reading pace, with higher (pronoun) skipping rates as a result. Obviously, this line of reasoning is speculative since a definitive answer requires a controlled examination of these variables. However, the point being made here is that previous reading studies may have underestimated ‘normal’ pronoun skipping rates in Dutch, rather than the current study overestimating these rates.

### Implications for the Risky Reading Hypothesis

In the present study readers that displayed long saccades and many regressions were more inclined to use verb-based implicit causality information to anticipate an upcoming referent, and consequently slowed down immediately when they encountered a pronoun that went against the bias of the verb. This pattern of results is consistent with the risky reading hypothesis (Rayner et al. 2006, 2009) in which longer saccades and more regressions are taken to be indicative of a proactive reading strategy. Moreover, the results suggest that the framework on risky reading, which was originally construed around the reading profiles of senior adult readers, can also be applied to younger adult readers.

An interesting line of future research is to further identify the characteristics of both older and younger adult readers that enforce a proactive reading strategy. Since reading is a complex skill, encompassing a large array of perceptual, linguistic and general cognitive mechanisms, it is evident that numerous factors will play a role. At the same time, previous studies have indicated that two factors will be of particular interest, namely the perceptual span size (the region of effective vision) and working memory capacity of readers.

Rayner et al. (2006, 2009) suggested that older adult readers resort to risky reading due to the deteriorating effects of aging on visual perception. They observed that older readers have a smaller perceptual span and therefore acquire less information during an eye-fixation, which slows down the reading process. Rayner et al. reasoned that older readers tend to anticipate (or even guess) the upcoming information of a text more often than younger readers to partially compensate for their slower processing of texts.

A different—yet, not necessarily incompatible – account has been put forward by Miller and Stine-Morrow (1998; see also Rayner et al. 2006). They suggest that older readers are not using alternative reading strategies to compensate for a declining perceptual span, but rather, to compensate for a diminishing working memory capacity. Their hypothesis is that older adults break up texts into smaller conceptual units than younger adults do, to avoid a resource overload of their working memory system. As a result, older adults allocate more processing resources to the integration of information as it is introduced in text, whereas younger adults tend to wait until the end of the sentence to fully integrate the information.

An important open issue is to what extent these accounts on the source of the reading strategies of older adult readers bear on the strategies of younger adult readers. That is, although at some level of analysis the proactive readers in the current study display similar eye-movement behaviour as senior adult readers, they may be doing so for very different reasons. For example, if we take the idea of Miller and Stine-Morrow (1998), and extrapolate from older readers to our two main groups of proactive and conservative young adult readers, it follows that the proactive readers should have a smaller working memory capacity than the conservative readers. This would explain the highly incremental reading strategy of our proactive readers—a strategy in which they anticipate and immediately integrate the (upcoming) information of a text.

Some previous studies on younger adult readers, however, seem inconsistent with this account. In particular, Long and De Ley (2000) showed that the activation of implicit causality information depends on a complex causal inference and is used only by highly-skilled readers in the middle of an unfolding sentence. In contrast, less-skilled readers lack the required working memory resources to deploy implicit causality information in mid-sentence, and only display a sensitivity to the implicit causality cue near the end of a sentence. Furthermore, comparable results were obtained in a recent eye-tracking study on garden-path sentences (Malsburg and Von der Vasishth 2013). In this study, high working memory capacity readers were making structural commitments in the middle of a sentence, yet lower working memory capacity readers were delaying or otherwise not forming those commitments. The latter two studies have in common that they both show that high working memory span readers use the information of a sentence right at the moment it becomes available, whereas lower working memory span readers use it in a delayed manner (e.g., as the result of a good-enough processing strategy, Ferreira and Patson 2007; see Malsburg and Von der Vasishth 2013). This seems to be inconsistent with, if not the exact opposite of, the hypothesis of Miller and Stine-Morrow (1998): they proposed that a lower or a diminishing working memory capacity triggered a more incremental processing strategy for senior adult readers in comparison to young adult readers.

In all, based on the evidence available at the present time, we can merely conclude that the relationship between working memory capacity and the reading strategies it promotes, is not always straightforward. This not only highlights the necessity to further disentangle the influence of working memory capacity on reading strategies, but in addition, it highlights the necessity to examine this interplay together with the influence of other readers’ characteristics. That is, the reading strategy of people will eventually be shaped by a (complex) interaction of several perceptual abilities (e.g., the perceptual span size), as well as linguistic and cognitive abilities, and future studies should address the issue accordingly.

### Implications for the Implicit Causality Literature

In addition to providing new evidence in relation to the risky reading hypothesis, the present study presents some novel insights into a prevailing theoretical issue in the implicit causality literature: the focusing versus integration debate. Whereas proponents of the focusing account assume that implicit causality information is used in a proactive way to anticipate an upcoming referent, proponents of the integration account claim that implicit causality influences reading processes in a retroactive integration phase only (see e.g., Garnham et al. 1996; McDonald and MacWhinney 1995; Pyykkönen and Järvikivi 2010; Stewart et al. 2000). The present findings sketch a more complex picture in which both accounts can be correct, depending on the reader’s processing strategy. On the one hand, a group of readers (the proactive readers) displayed behavior that was consistent with the predictions of the focusing account (i.e., a reading time delay at the pronoun itself), signifying an anticipatory use of implicit causality information. On the other hand, there were also many readers (the conservative and fast readers) for whom the influence of implicit causality was delayed, suggesting that they were using implicit causality to resolve the pronoun retroactively instead (cf., Long and De Ley 2000).

Moreover, for the slow readers neither the focusing nor the integration account provides an adequate explanation. This group of readers did not notice—or did not use—implicit causality information during reading. In fact, they showed a reversed pronoun inconsistency effect. An interesting possibility is that this reversal of the effect is related to the phenomenon that in addition to an implicit causality bias, interpersonal verbs often exhibit an implicit consequentiality bias (Au 1986; Crinean and Garnham 2006; Garnham 2001; Koornneef and Sanders 2013; Rigalleau et al. 2014; Stewart et al. 1998). Implicit consequentiality is usually defined as a bias towards the argument of the verb that is affected by the consequence of an interpersonal event. Coincidentally or not (see Crinean and Garnham 2006), the majority of the verbs in our stimulus set show opposite implicit causality and consequentiality biases (e.g., ‘to fascinate’ has a strong implicit causality bias to NP1, but an equally strong implicit consequentiality bias to NP2; see Koornneef and Sanders 2013 for details and further discussion). Hence, it is possible that the pronoun resolution process of the slow readers is guided by the implicit consequentiality bias of verbs, whereas proactive, conservative and fast readers are more inclined to use the implicit causality bias.

If this explanation is correct, it would raise some interesting questions about the nature of implicit causality. For example, a dominant view on the source of implicit causality (and implicit consequentiality) is that the relevant causal information is stored in the lexical representation of the verb (for overviews see Crinean and Garnham 2006; Hartshorne and Snedeker 2013; Pickering and Majid 2007). Given the clear contrast between slow readers on the one hand, and proactive, conservative and fast readers on the other, this could imply that the lexical representation of implicit causality verbs may vary from reader to reader. In that sense an interesting parallel can be drawn with current developments in broader frameworks of reading. For example, Perfetti and Stafura (2014; see also Perfetti 2007; Perfetti and Hart 2002) explicitly state that reading entails linkage between a word identification system and a comprehension system, with the lexicon in the linking role. Furthermore, they argue that individual differences in reading emerge primarily as a function of the quality—both in form (orthographical, phonological) and meaning—of the lexical entries in the long-term memory systems of the reader. Although we do not want to go as far as suggesting that the slower readers have inferior lexical representations, the framework proposed by Perfetti and his colleagues reinforces the idea that the lexical entries of implicit causality verbs may diverge considerably across readers—thereby accounting for the contrasts as observed in the present study.

## Concluding Remarks

By investigating the saccadic eye movements of readers and linking the reading patterns to the time course of implicit causality, we were able to confirm and extend the implications of the risky reading hypothesis (Rayner et al. 2006, 2009). A general conclusion that can be drawn from this is that the eye-movement patterns of readers reveal important information about their processing strategy. In addition, the findings raise some intriguing questions on the mechanisms underlying the activation and usage of verb-based implicit causality. Most importantly, our results suggest that the real goal for future research is not to decide between focusing or integration accounts, but to decipher which factors determine the (proactive) use of implicit causality. We emphasize that a very important mediating factor will be the reader himself. Readers differ in terms of their cognitive and perceptual abilities, they use different processing strategies, and—more speculatively—the lexical entries of implicit causality verbs may vary across readers. Together these individual differences may largely determine whether (and when) implicit causality is used during comprehension.

Next to these issues pertaining to between-reader variation, there is also a question of potential within-reader variation. It is possible, perhaps even likely, that readers exhibit different reading profiles depending on the type of text they are reading, adapting their strategy to suit their current processing goals. The design of the current study does not allow us to shed light on the question of whether reading profiles are a general property of the reader or whether the same reader can exhibit different profiles depending on the situation and type of text they are reading, but it is an important question.

Future eye-tracking studies should be able to shed more light on the issues addressed above. However, although the development of the eye-tracking field has been impressive over the last decades (Radach et al. 2008; Rayner 1998) a crucial step needs to be made. Our study illustrates that it is time to combine the insights from two main research traditions that have evolved in the reading literature (cf., Drieghe et al. 2004, 2007). More specifically, psycholinguistic studies that use eye tracking to test hypotheses about language comprehension (e.g., at the syntactic, semantic and discourse level) should not ignore the literature aiming at the development of eye-movement control models that describe the more low level, visuo-motor characteristics of eye-movement behavior (e.g., the distance of saccades). And similarly, scholars in the latter tradition should not be blind to the influence of (psycho)linguistic factors on eye-movement control (cf., Radach et al. 2008; Rayner et al. 2006, 2009). Together, these fields allow a more thorough examination of reading processes in general, and an in-depth analysis of the individual differences between readers in particular.

## Footnotes

1 For reasons of clarity we created the labels *fast* and *slow* for the remaining groups—as opposed to referring to them solely on the basis of saccade distance and probability of regressions. However, note that these labels are, of course, somewhat arbitrarily chosen.

2 For a discussion of the same data set, but collapsed over all groups, see Experiment 1 of Koornneef et al. (2015).

3 The statistical models reported in the current study were estimated with the lme4 R package (R version 3.1.2, lme4 version 1.1.7).

4 Unless mentioned otherwise, the regression formulas in the Exploratory Analyses section were as follows: Dependent Measure $=$  1 $+$ Consistency $+$ (1 $+$ Consistency | subject) $+$ (1 $+$ Consistency | item).

5 To obtain convergence, a reduced complexity of the item random-effect structure was required for the first three models reported for the slow readers: Dependent Measure = 1 $+$ Consistency $+$ (1 $+$ Consistency | subject) $+$ (1 | item).

6 The regression formula: Dependent Variable = 1 + Group $\times$ Consistency $+$ (1 | participant) $+$ (1 | item).

7 As pointed out to us by an anonymous reviewer, examining classical reading time measures (i.e., first fixation duration, regression path duration etc.) at a confined region immediately preceding the main region of interest (i.e., in our case the pronoun) may not be the best approach to capture parafoveal preview effects. Another way is to examine the duration of the last fixation prior to a pronoun being skipped. We also conducted these analyses, yet the results mirrored the results discussed above. That is, neither an effect of Consistency, nor a Consistency $\times$ Group interaction emerged at the last fixation prior to skipping a pronoun.

8 We also conducted a different type of ‘contingent’ analyses for the proactive readers, in which we looked at the duration of the first fixation that followed the saccade during which the pronoun was being skipped. This type of analysis focuses somewhat more on the order of fixations, rather than on static regions of interest (cf., footnote 7). Therefore, it is potentially better suited to capture very early inconsistency effects in trials where the pronoun has been skipped (i.e., since it allows for more flexibility regarding the landing site of the first post-pronoun fixation). However, as it turns out, in the current case the mixed-effects analysis on the durations of these first post-pronoun fixations did not reveal a significant effect of Consistency for the proactive readers ($M_{\mathrm{con}}= 222\,\mathrm{ms}, {\textit{SE}}_{\mathrm{con}} = 6\,\mathrm{ms}; M_{\mathrm{inc}}= 218\,\mathrm{ms}, {\textit{SE}}_{\mathrm{inc}}= 5\,\mathrm{ms}$).

9 The skipping rates as a function of reading group: Proactive readers, Block 1 = 88 %, Block 4 = 83 %; Conservative readers, Block 1 = 85%, Block 4 = 77%; Fast readers, Block 1 = 86 %, Block 4 = 83 %; Slow readers, Block 1 = 83 %, Block 4 = 87 %.
